# Hot Deformation Behavior of a Hot-Isostatically Pressed Ti-6Al-4V Alloy from Recycled Powder

**DOI:** 10.3390/ma17050990

**Published:** 2024-02-21

**Authors:** Ruili Guo, Naixu Wang, Min Cheng

**Affiliations:** 1 School of Civil Engineering and Architecture, Wuhan Institute of Technology, Wuhan 430074, China; 20120103@wit.edu.cn; 2 College of Materials Science and Engineering, Taiyuan University of Technology, Taiyuan 030024, China

**Keywords:** hot deformation behavior, processing maps, powder metallurgy, powder reuse, Ti-6Al-4V alloy

## Abstract

In this work, a new use of mixed Ti-6Al-4V powder, consisting of the retained powder after screening for additive manufacturing and the recycled powder after multiple printing, has been exploited. The powder mixture has been hot-isostatically-pressed (HIPed) at 930 °C/120 MPa for 3 h to reach full density. The hot deformation behavior of the as-HIPed powder compacts were investigated through isothermal compression tests, kinetic analyses, and hot processing maps. Finally, the optimized hot working parameters were validated using upsetting tests. The results show that the as-HIPed Ti-6Al-4V alloy has a fine and homogeneous microstructure. The activation energies were calculated to be 359 kJ/mol in the α + β phase regime and 463 kJ/mol in the β phase regime, respectively. The optimal hot working parameters are a deformation temperature above 950 °C and strain rate higher than 0.1 s^−1^. The hot workability of as-HIPed powder compacts is better than the as-cast billets. The deformed microstructure can be finer than that of as-HIPed state, and the mechanical performance can be further improved by the optimal thermo-mechanical processing treatment.

## 1. Introduction

Titanium and titanium alloys have been widely used in aerospace, petrochemicals, marine vessels, and biomedical fields because of their high specific strength and stiffness, excellent fatigue and corrosion resistance, and outstanding biocompatibility [1,2,3]. However, their high production costs limit the further application of titanium alloys in our daily life [4,5]. This is because the extraction and machining costs of titanium alloys are much higher than those of the traditional steel and aluminum alloys. The common processing methods for Ti-based alloy components are casting and forging. The low superheat temperature of titanium alloy melt makes it difficult for the mold-filling process. Additionally, the melt of titanium is very active, and it can react with almost all crucible materials [6,7]. In order to improve the castability of titanium melt, filed-assisted casting techniques have been developed to fabricate near-net-shape parts with complex structures. However, their coarse microstructure and inevitable casting defects such as porosity, shrinkage, and ceramic inclusions can worsen the properties of the cast alloys and/or components [6,8,9]. Feng et al. [10] reported that large-size Ti-6Al-4V thin-wall casing could be successfully fabricated by centrifugal casting, and the effects of mold pre-heating temperatures and the hot isostatic pressing (HIP) process on the microstructural characteristics and mechanical properties. With the increase of the mold pre-heating temperature, the porosity of the large-size thin-wall structure decreases obviously. HIP is also an effective way to heal the residual porosity and improve the ductility of as-cast Ti-based alloys. The as-forged titanium alloys have excellent mechanical properties, and their microstructure can be precisely regulated by thermo-mechanical processing and subsequent heat treatments [11,12,13,14]. Chouhan et al. [15] reported that an equiaxed nanostructure, optimized dislocation substructure, and prismatic fiber texture can be obtained by multiaxial plane-strain forging and rolling, and the mechanical properties, tensile strength, and toughness can be significantly improved. Careau et al. [16] reported that direct powder forging (DPF) has significant potential as an alternative fabrication strategy for the consolidation and in situ alloying of Ti-based alloys. However, the utilization of the as-forged billet to the final complex component may be very low, even only about 8–10% for the engine parts in F22 [17].

Recently, additive manufacturing (AM) that produces metallic parts layer-by-layer has been considered as a revolutionary technique, which has a very important impact on metallurgical research [18,19,20]. Among all AM techniques, laser powder bed fusion (LPBF) and electron beam melting (EBM) have aroused significant attention [21,22,23]. These methods can produce complex components and achieve near-net-shape forming. However, the oxygen content of the powder will increase after multiple printing, and other powder characteristics will also change. Up until now, the reuse of the powder is still a challenge [24,25,26]. At the same time, the optimal particle size distributions of titanium alloy powders for LPBF and EBM are 5–53 μm and 45–105 μm. As is known, the particle size distribution of Ti-based alloy powder is in the range from 5 to 250 μm, such as that of the gas-atomized powders [27,28]. The utilization of the sieved powders after screening for AM is also an important issue to be exploited. Soltani-Tehrani et al. [29] reported that there is only minimal influence of limited reuse of recycled powder on the performance of SLM Ti-6Al-4V products. However, the changes in oxygen content, powder surface morphology, and particle size distribution for the recycled powder can obviously influence the actual applications of powder reuse [30]. Guo et al. [31] reported that it is feasible to fabricate large-size and high-performance titanium alloy rings using sieved coarse powders with a size range of 53–250 μm. Therefore, this work aims to exploit the recycling of the powder mixture consisting of sieved powder after screening for SLM and the recycled powders after multiple printing. The Ti-6Al-4V powder mixtures were HIPed at 930 °C/120 MPa for 3 h. Subsequently, the hot deformation behavior of as-HIPed powder compacts were studied through isothermal compression tests, kinetic analyses and processing maps. Lastly, an upsetting test was conducted to valid the optimized hot compression parameters based on the processing map.

## 2. Materials and Methods

### 2.1. As-Received Powders

**Figure 1:**
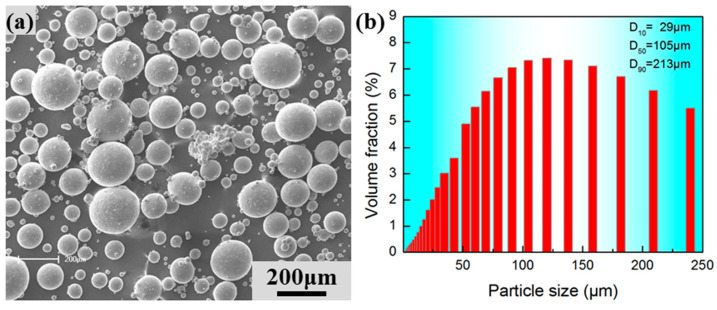
(**a**) Powder morphology and (**b**) particle size distribution of the mixed Ti-6Al-4V powder.

The Ti-6Al-4V powder was made via an electrode-induced gas atomization (EIGA) process using a crucible-free system. The chemical composition of the as-received powder, defined as PI, is shown in Table 1. It is noted that the oxygen content of PI was 0.09%. The P1 powder was sieved into two kinds of powders with different particle size distributions. The first is the fine power with the size range of 5–53 μm, defined as PII, which was produced for LPBF. The second is the retained coarse powder with the size range of 53–250 μm, defined as PIII. In the present work, two kinds of Ti-6Al-4V powders, the recycling PII powder after five times (5×) LPBF (defined as PIV) and the PIII powder, were used. The PIII and PIV powders were filled into tanks with the weight ratio of 4:6. The mixed powders were milled for 4 h with a speed of 150 r/min and a ball-to-material ratio of 5:1 through a SFM-1 ball mill. The mixture of PIII and PIV powders was defined as PV. The chemical composition of this powder mixture (PV) is also shown in Table 1. It was found that the oxygen content of the PIV powder is 0.13%, which was a little higher than that of the PI powder. It was indicated that the PII powder would pick up oxygen during the multi-printing. The powder morphology and particle size distribution are shown in Figure 1. It was found that the powder particles were nearly spherical and the mean particle size is about 105 μm. It was noted that some irregular powders could be observed in the PV powder, and that multi-printing may change the powder surfaces.

### 2.2. Experimental Procedures

**Figure 2:**
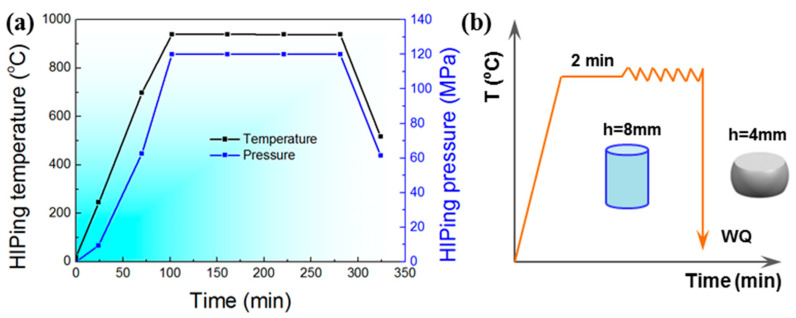
Schematic diagrams of (**a**) the HIP procedure, and (**b**) the isothermal compression test.

The PV powder was filled in thin-wall mild-steel containers, and then HIPed at 930 °C/120 MPa for 3 h (Figure 2a). The HIP temperature and pressure were raised at the same time. The heat rate was about 5 °C/min. The dimensions of as-HIPed powder compacts included a diameter of 60 mm and a height of 120 mm. Samples with dimensions of 8 mm in diameter and 12 mm in height were cut from the HIPed powder compacts, and the hot compression tests were performed using a Gleeble 3800 thermal simulator (Dynamic Systems Inc., Albany, NY, USA). The as-machined surface finish was used. The testing temperature was set as 800–1000 °C, and the strain rate was set as 0.01–10 s^−1^. The specimens were heated to the selected temperature and dwelled there for 2 min before compression (Figure 2b). All of the samples were compressed to the half of their height, and the maximum true strain was about 0.7. After the hot compression, the deformed specimens were water-quenched immediately to preserve the high-temperature microstructures. The true stress–strain curves were automatically recorded during the compression process. The deformed specimens were sectioned parallel to the compression axis through the center to observe the microstructural evolution. The dimensions of the specimen used for the metallographic observation were 6 mm × 6 mm × 3 mm. After being ground, polished, and etched using the Kroll agent (4 mL HNO_3_, 8 mL HF and 88 mL H_2_O), the microstructures of the deformed specimens were characterized with DM4000M optical microscopy (OM). The dog-bone bar tensile specimens were cut from the deformed Ti-6Al-4V billets after upsetting tests. The diameter and gauge length of the tensile specimens were 3 mm and 15 mm, respectively. The tensile tests were carried out on an INSTRON 5969 testing machine, and the strain rate was 10^−3^/s. Three to five tensile specimens were used for each condition.

## 3. Results and Discussion

### 3.1. Microstructures of As-HIPed Ti-6Al-4V

**Figure 3:**
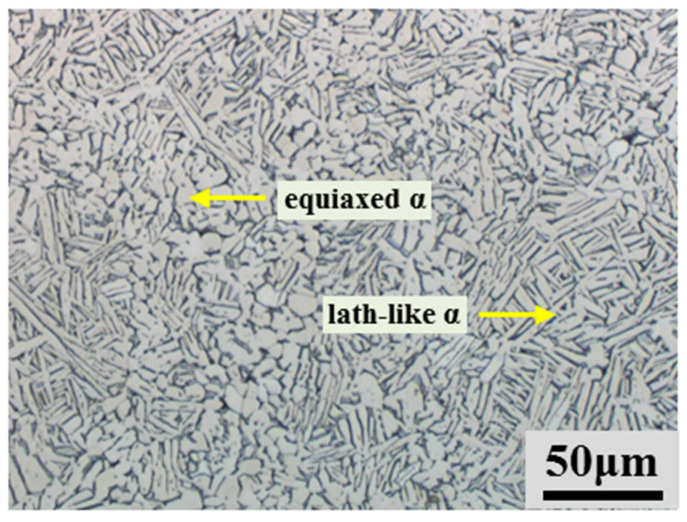
OM image of an as-HIPed Ti-6Al-4V billet.

Figure 3 depicts the microstructure of an as-HIPed Ti-6Al-4V billet before hot compression tests. The microstructure that is made up of equiaxed and lath-like α phases and β lamellae is fine and homogenous, and no obvious porosity can be observed. The relative density of as-HIPed billets is approaching full density. The grain size of the equiaxed α phase is ~7.8 μm and the width of the lath-like α phase is ~2.2 μm. It is easy to understand that an oxide layer on the powder surface may be formed after multi-printing. However, there are no obvious defects such as oxide inclusion and prior particle boundaries in the as-HIPed Ti-6Al-4V billet. The HIP process of the encapsulated powders can be divided into four stages: particle movement and rearrangement, plastic deformation, creeping, and high-temperature diffusion. Obviously, the fine particles can occupy the voids among the coarse particles during the stage of particle rearrangement [32]. In addition, the powder surfaces of the fine powder would also go through severe plastic deformation, resulting in the breakage of the oxide layer. When the powder compacts were heated to the desired HIP temperature, the oxygen would diffuse from the oxide particle to the titanium matrix, leading to the elimination of oxides. At the same time, equiaxed grains will be formed due to the plastic deformation and dynamic recrystallization (DRX) around the powder particles’ boundaries [33,34].

### 3.2. Isothermal Compression Tests

#### 3.2.1. Stress–Strain Curves

**Figure 4:**
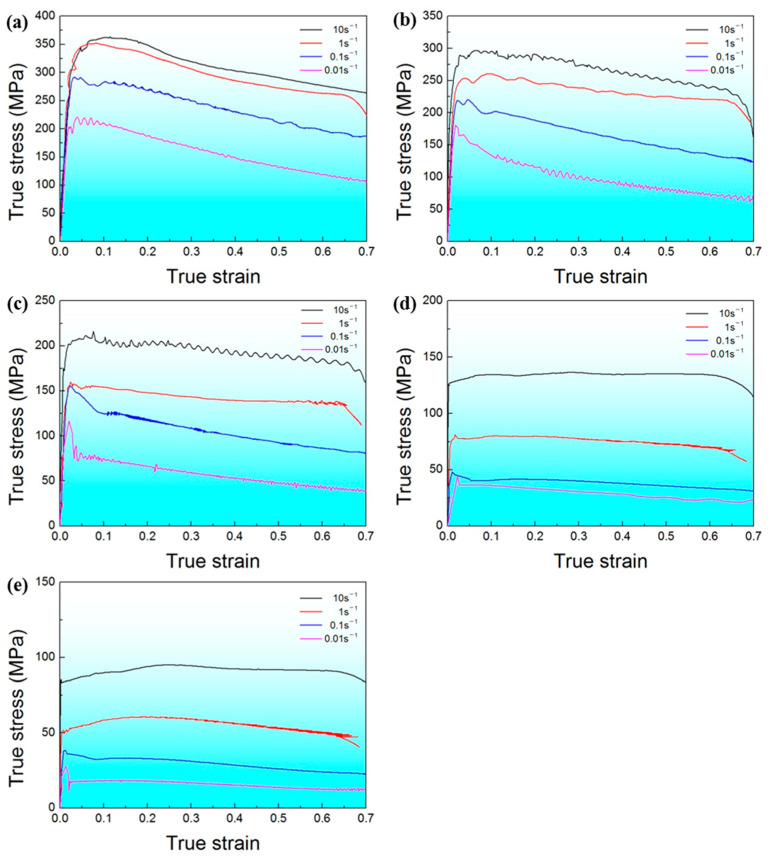
True stress–strain curves of as-HIPed Ti-6Al-4V billets deformed under various temperatures and strain rates: (**a**) 800 °C, (**b**) 850 °C, (**c**) 900 °C, (**d**) 950 °C, (**e**) 1000 °C.

Figure 4 depicts the true stress-true strain curves of Ti-6Al-4V billets under various deformed temperatures and strain rates. When the deformation temperature is below 850 °C, the deformation behavior of the Ti-6Al-4V alloy shows a trend of hardening first and then softening, which is caused by the competition between dynamic softening and work hardening during the compression process. In the early stage of hot compression deformation, the Ti-6Al-4V alloy is mainly composed of α-Ti, similar to the microstructure shown in Figure 3. The accumulation of dislocations results in a high work hardening rate and the work hardening becomes more pronounced with the increase in deformation rate [35,36]. However, when the deformation reaches a specific value, the flow stress gradually decreases with the increasing deformation, and the dynamic softening behavior dominates the subsequent deformation. When the deformation temperature is 900 °C (low α + β phase regime), the deformation behavior of the Ti-6Al-4V billet still exhibits the trend of hardening first, then softening at lower deformation rates. However, at higher deformation rates, the deformation behavior of the samples is similar to that at 950 °C (high α + β phase regime) and 1000 °C (β phase regime). The deformed samples show hardening first and then steady-state plastic flow behavior.

**Figure 5:**
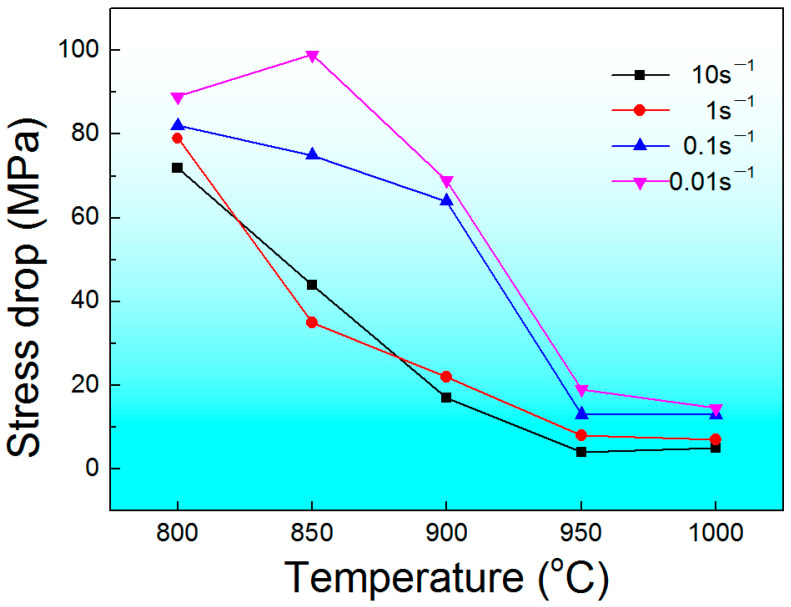
The stress drop of Ti-6Al-4V billets under various compression temperatures and strain rates.

Flow softening was observed for the compressed samples at all temperatures. Previous work reported that the flow softening was related to the adiabatic heating effect, resulting in the temperature rise and DRX [37]. In this study, in order to elucidate the dynamic flow softening behavior of as-HIPed Ti-6Al-4V billets, the stress drop (∆*σ*) was expressed as follows [38]:

∆*σ* = *σ*_p_ − *σ*_0.7_ (1)

where *σ*_p_ and *σ*_0.7_ are the peak stress and the flow stress at a true strain of 0.7. Figure 5 depicts the stress drop of a Ti-6Al-4V billet at all compression temperatures and strain rates. The stress drop is larger at the lower temperatures and higher strain rates. The flow softening can be mainly caused by the following two reasons: First, with the increase in adiabatic temperature and deformation time, the volume fractions of the β phase in the compressed samples increase. However, the β phase is not conducive to the accumulation of dislocations at grain boundaries compared with the α phase. Therefore, the stress drop under a low compressed temperature and low strain rate is larger. Second, dynamic recovery and DRX may occur during the deformation process, which may also reduce the work hardening.

#### 3.2.2. Kinetic Analysis

The true stress–strain curve indicates that strain rate and deformation temperature have a significant impact on flow stress. Peak stress can be chosen as the representative stress of each strain–stress curve to reveal the relationships between flow stress, strain rate, and deformation temperature (Figure 4). The peak stress of compressed samples increases with the increasing strain rate and decreasing deformation temperature. It is also worth noting that the slops of the *m*-value plots in the α + β phase regime is different from that in the β phase regime, indicating that different deformation mechanisms dominate under different compression conditions. Therefore, the relationships among the flow stress, the deformation temperature, and the strain rate during the isothermal compression can be expressed as follows [39]:

(2) $$\dot{\varepsilon }={A[sinh(\alpha_{1}\sigma_{p})]}^{n}exp[-\frac{Q}{RT}]$$

where *A*, *R*, *α*_1_, and *n* are constants, *Q* is the activation energy, and $\dot{\varepsilon }$ is the strain rate. It is expected that the hot deformation mechanism of Ti-6Al-4V billet is determined by the *Q*-value and microstructure. The *Q*-value can be calculated using the following equation [40]:

(3) Q=−nR∂ln⁡ε˙∂(1/T)=σR [d lnε˙d ln⁡(σ)]T[d ln⁡(σ)d (1/T)]ε˙

**Figure 6:**
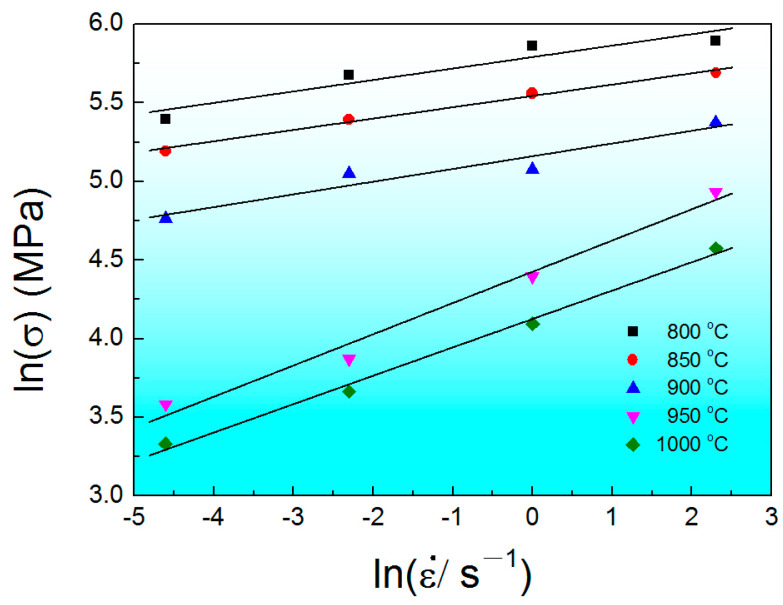
Relationship between ln (*σ*) and ln ($\dot{\varepsilon }$).

Figure 6 depicts the variation in flow stress with strain rate. In Equation (3), the *Q*-value can be calculated from the slopes of the linear relationship between ln (*σ*) and ln ($\dot{\varepsilon }$) and between ln (*σ*) and 1/*T* with the help of the stress–strain curves. As for the as-HIPed Ti-6Al-4V billet, the *Q*-value obtained in the α + β phase regime is 359 kJ/mol and is 463 kJ/mol in the β phase regime, which are much larger than the self-diffusion activation energy for Ti/Al and some other titanium alloys [41]. This indicates that besides the self-diffusion mechanism, other deformation mechanisms such as DRX could also contribute to the deformation process.

#### 3.2.3. Processing Maps

As is known, processing maps established through the dynamic materials model [42] have been considered as an effective way to reveal hot deformation mechanisms and are beneficial to optimize hot working parameters and regulate the microstructure in titanium alloys. In the present work, a processing map has been established to investigate the deformation behavior of HIPed Ti-6Al-4V billets at different temperatures. In the DDM model, the principle is to combine the work carried out by external forces on the material and the energy consumed by plastic deformation. The total absorption power (*P*) can be defined as follows:

(4) $$P=\sigma \dot{\varepsilon }=G+J=\int_{0}^{\dot{\varepsilon }}\sigma d\dot{\varepsilon }+\int_{0}^{\sigma }\dot{\varepsilon }d\sigma$$

where *G* represents the consumed energy due to plastic deformation at high temperatures, which can be considered as the thermal energy transformed from the kinetic energy of atoms. *J* is the consumed energy due to microstructure changes during deformation. The ratio of *J*- and *G*-values can be represented by the coefficient *m*, which is also called the strain rate sensitive index. Ideally, when the energy dissipation of the system is linear energy dissipation, the *m*-value is equal to 1 and the *J*-value achieves the maximum *P*/2. The *m*-value is also an important variable parameter to establish the processing maps. And the *m*-value can be expressed with the following equation [43]:

(5) $$m=\frac{\partial \ln\sigma }{\partial \;ln\dot{\varepsilon }}$$

The efficiency of power dissipation (*η*), can be defined as follows [44]:

(6) $$\eta =\frac{J}{J_{max}}=\frac{2m}{m+1}$$

In Equation (5), it is obvious that the *m*-value is affected by the strain rates and the deformation temperatures. Generally speaking, the *m*-value increases with the increase in deformation temperature because the increasing deformation temperature will accelerate the grain boundary sliding and enhance the diffusion rate [43]. The variation in the *η*-value with the deforming temperature and strain rate constitutes the power dissipation map.

According to the extremum principle of alloys during large-strain plastic deformation, the criterion for material flow instability can be obtained as follows [45]:

(7) $$\xi \left(\dot{\varepsilon }\right)=\frac{\partial lg\frac{m}{m+1}}{\partial lg\dot{\varepsilon }}+m<0$$

where $\xi \left(\dot{\varepsilon }\right)$ is a dimensionless instability parameter. When the value of $\xi \left(\dot{\varepsilon }\right)$ is negative, flow instability is expected to occur, and the unsafe processing domains can be identified.

**Figure 7:**
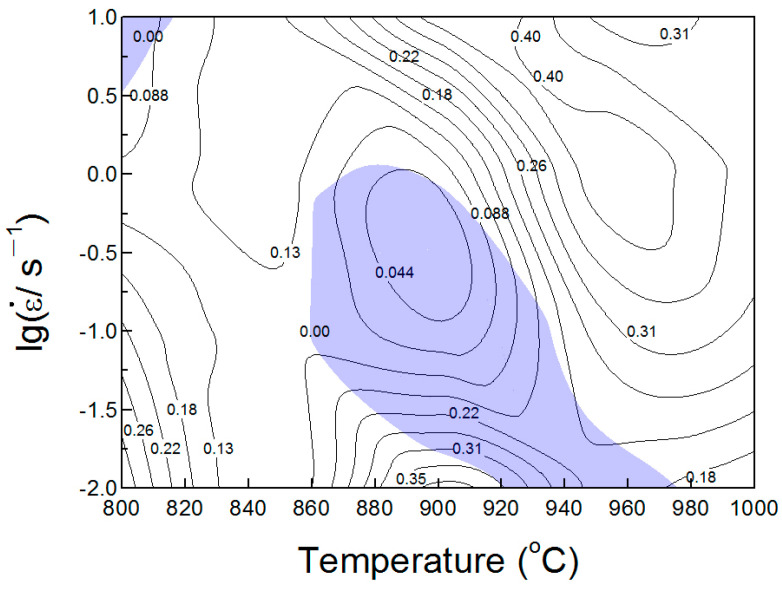
Processing map of the as-HIPed Ti-6Al-4V alloy at the true strain of 0.7.

The plastic flow instability map presents the variation in instability with the strain rate and deforming temperature. The processing map is a combination of the superimposition of the power dissipation map and flow instability map. The optimized hot working parameters can be designed and a favorable microstructure and the corresponding properties can be obtained based on the processing maps. Figure 7 depicts the processing map of the HIPed Ti-6Al-4V billet at a true strain of 0.7. The values of the contour lines with numbers represent the powder dissipation factor *η*. The area marked in blue represents the flow instability zone, while the other areas are safe zones. The larger the *η*-value, the more favorable it is for the compression processing of the Ti-6Al-4V billet in the safe zone. However, almost all of the input energy is converted into the energy used for plastic deformation, making it prone to cracking and unsuitable for hot working in the flow instability zone. When the deformation temperature is above 950 °C, and the strain rate more than 0.1 s^−1^, the *η*-value is greater than 0.3. This indicates that this area is the optimal deformation zone. In addition, when the deformation temperature is at 900 °C and the strain rate is 0.01 s^−1^, although the *η*-value is also larger than 0.3, this area is closer to the flow instability zone. At the same time, when the temperature is below 880 °C, the *η*-value decreased significantly. Therefore, the preferred hot deformation window for the HIPed Ti-6Al-4V billet in this study is: a deformation temperature above 950 °C, and a stain rate > 0.1 s^−1^. Compared with as-cast Ti-6Al-4V billets [46], the as-HIPed powder compacts have better hot workability. This may be due to the microstructure of the as-HIPed Ti-6Al-4V billet being finer and more homogenous [31,47].

### 3.3. Deformed Microstructure

**Figure 8:**
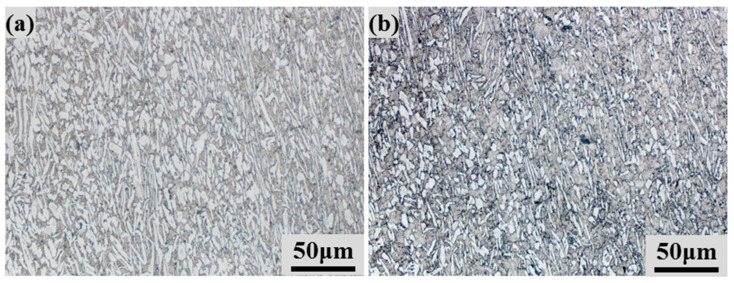
As-deformed microstructures of as-HIPed Ti-6Al-4V powder compacts at different compression temperatures with the strain rate of 10 s^−1^: (**a**) at 900 °C; (**b**) at 1000 °C.

Figure 8 depicts the as-deformed microstructure of the as-HIPed Ti-6Al-4V billet at 900 °C and 1000 °C under the strain rate of 10 s^−1^. The compressed grains have been elongated, and the as-deformed microstructure consists of primary α (white) and transformed β (grey) phases. In Figure 8b, it can be seen that the grain size of the equiaxed α phase is about 2.8 μm, and the width of the α lamellae is about 0.8 μm. Compared with the as-HIPed microstructure in Figure 3, the grain size of this as-compressed microstructure can be much finer at high strain rates. It is believed that the grain size and volume fraction of the primary α phase can be obtained by altering the hot deformation conditions and it is expected that the mechanical performance of powder compacts can be further improved after optimal hot deformation parameters.

### 3.4. Upsetting Tests

**Figure 9:**
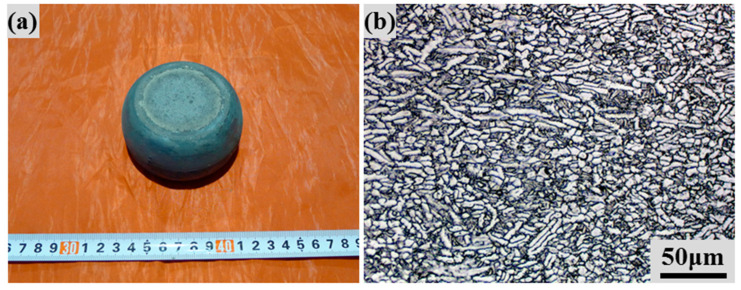
Picture (**a**) and OM image (**b**) of as-deformed Ti-6Al-4V billet.

The as-HIPed powder compacts (60 mm in diameter and 120 mm in height) were used for the upsetting tests to validate the optimized hot compression parameters based on the processing map. Considering there is a temperature drop during the upsetting tests due to heat loss to the surroundings, an upsetting temperature of 980 °C and a strain rate of ~1 s^−1^ were used. After upsetting, no obvious cracks can be observed at the edges of the Ti-6Al-4V billet in the present study. Figure 9 depicts a picture and the microstructure of the as-deformed power compacts after upsetting. Although the samples were heated to 980 °C, the upsetting time is very short, and the kinetic growth of the grain size is restricted. However, the high strain rate will enlarge the strain energy of the powder compacts, and the grain may be elongated and refined due to dynamic recrystallization (DRX) during the deformation and the following cooling stage [43,47]. Therefore, the as-deformed microstructure is finer than the as-HIPed samples. It is also worth noting that the volume fraction of equiaxed grains increases after upsetting. A comparison of the tensile properties of the as-HIPed and as-deformed powder compacts is shown in Table 2. The upsetting compression tests can increase the tensile strength due to the finer microstructure with no sacrifice of the tensile ductility. This indicates that the hot workability of as-HIPed Ti-6Al-4V powder compacts is better than that of as-cast alloys, and that better tensile properties can be achieved after optimal thermomechanical processing treatments.

## 4. Conclusions

In this paper, Ti-6Al-4V powder compacts were HIPed using the mixed powder by the retained powder after screening for SLM and the recycled powder after five iterations of SLM. The hot deformation behavior of as-HIPed powder compacts was investigated and upsetting tests were conducted as the validation experiments. The following conclusions have been drawn:

(1) The as-HIPed microstructure of Ti-6Al-4V powder compacts is fine and homogenous. The retained powder after screening for SLM and the recycled powder after SLM can be used to fabricate the powder compact by the HIP process.
(2) The flow stress is sensitive to the strain rate and the deformation temperature. The activation energies are calculated to be 359 kJ/mol in the α + β phase regime and 463 kJ/mol in the β phase regime, respectively. Based on the processing map of the as-HIPed billets, the optimal hot working parameters are a deformation temperature above 950 °C and a strain rate higher than 0.1 s^−1^.
(3) The tensile properties of the as-deformed samples (YS-872 MPa, UTS-976 MPa) are better than those of the as-HIPed powder compacts (YS-842 MPa, UTS-930 MPa). The hot workability of as-HIPed powder compacts is better than that of as-cast Ti-6Al-4V billets, and the desired microstructure and mechanical properties can be achieved using the optimal thermo-mechanical processing treatment of powder compacts in the present work.

## Figures and Tables

**Table 1:** Chemical composition of Ti-6Al-4V powder mixtures.

Samples	Al	V	Fe	C	O	N
PI	6.1	4.0	0.14	0.03	0.09	0.01
PV	6.2	3.9	0.15	0.05	0.13	0.01

**Table 2:** Tensile properties of as-HIPed and as-deformed Ti-6Al-4V billets.

Samples	YS	UTS	El.	A
As-HIPed	842 ± 3	930 ± 1	15.7 ± 0.2	39 ± 1
As-deformed	878 ± 1	976 ± 2	15.5 ± 0.3	43 ± 2

Note: YS—yield strength, UTS—ultra tensile strength, El.—elongation, A—reduction in area.

## Data Availability

Data will be made available on request.
